# Sexually dimorphic response of mice to the Western‐style diet caused by deficiency of fatty acid binding protein 6 (Fabp6)

**DOI:** 10.14814/phy2.14733

**Published:** 2021-02-01

**Authors:** Salam M. Habib, Brittnee L. Zwicker, Linda Wykes, Luis B. Agellon

**Affiliations:** ^1^ School of Human Nutrition McGill University Montreal QC Canada; ^2^ Present address: McGill University Health Centre Montreal QC H4A 3J1 Canada

**Keywords:** bile acid malabsorption, fat malabsorption, fatty acid binding protein, gut microbiota, obesity, sexual dimorphism

## Abstract

Bile acids are natural detergents that aid in the absorption of dietary lipids. Fatty acid binding protein 6 (Fabp6) is a component of the bile acid recovery system that operates in the small intestine. The aim of this study was to determine if Fabp6 deficiency causes dietary fat malabsorption. Wild‐type and Fabp6‐deficient mice were fed a Western‐style diet (WSD) or a reference low‐fat diet (LFD) for 10 weeks. The body weight gain, bile acid excretion, fat excretion, energy metabolism, and major gut microbial phyla of the mice were assessed at the end of the controlled diet period. *Fabp6* ^−/−^ mice exhibited enhanced excretion of both bile acids and fat on the WSD but not on the LFD diet. Paradoxically, male *Fabp6* ^−/−^ mice, but not female *Fabp6* ^−/−^ mice, had greater adiposity despite increased fat excretion. Analysis of energy intake and of expenditure by indirect calorimetry revealed sex differences in physical activity level and respiratory quotient, but these did not account for the enhanced adiposity displayed by male *Fabp6* ^−/−^ mice. Analysis of stool DNA showed sex‐specific changes in the abundance of major phyla of bacteria in response to Fabp6 deficiency and WSD feeding. The results obtained indicate that the malabsorption of bile acids that occurs in *Fabp6* ^−/−^ mice is associated with dietary fat malabsorption on the high‐fat diet but not on the low‐fat diet. The WSD induced a sexually dimorphic increase in adiposity displayed by *Fabp6* ^−/−^ mice and sexually distinct pattern of change in gut microbiota composition.

## Graphical abstract

Loss‐of‐function of Fabp6 in mice can cause fat malabsorption when fed a Western‐style diet, sex‐specific changes in the composition of gut microbiota and sexually dimorphic body weight gain.

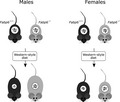

## 1 INTRODUCTION

Bile acids are important in facilitating the digestion and absorption of dietary lipids and lipid‐soluble molecules (Agellon, 2008). Bile acids are produced in the liver from cholesterol and released into the lumen of the small intestine upon meal ingestion. Gut bacteria are capable of metabolizing bile acids, transforming them into metabolites known as secondary and tertiary bile acids. The majority of bile acids are reabsorbed by the ileum, returned to the liver via the portal vein, and then stored in the gallbladder or resecreted into the small intestine as needed (Agellon, 2008). Bile acids that are not recovered by the ileum are passed to the large intestine and eventually excreted.

In ileal enterocytes, the mechanism for the recovery of bile acids consists of three main components: the apical sodium‐dependent bile acid transporter (Asbt; murine gene symbol *Slc10a2*), the heteromeric basolateral efflux transporter organic solute transporter (comprised of Ostα and Ostβ; murine gene symbols *Slc51a and Slc51b*, respectively), and the intracellular bile acid binding protein Fabp6 (Fabp6, also known as ileal lipid binding protein; murine gene symbol *Fabp6*) (Zwicker & Agellon, 2013). In mice, gene variations that cause loss‐of‐function of any of these components result in bile acid malabsorption (BAM; Ballatori et al., 2008; Dawson et al., 2003; Oelkers et al., 1997; Praslickova et al., 2012; Rao et al., 2008).

In humans, mutations in the *SLC10A2* gene cause primary BAM (Oelkers et al., 1997). BAM can be also result from ileal resection, or secondary to digestive tract disorders such as irritable bowel syndrome, pancreatic insufficiency, and celiac disease (Watson et al., 2015). In general, BAM is characterized by excessive amounts of bile acids passed into the colon, where it causes enhanced electrolyte and water excretion and chronic steatorrhea. Fat malabsorption was not observed in mice lacking Asbt (Dawson et al., 2003), however, this may be due to efficient intestinal fat absorption in this species. In this study, we evaluated the relationship between BAM and fat excretion in the context of dietary fat content in mice with and without Fapb6.

## 2 MATERIALS AND METHODS

### 2.1 Mice and diets

C57BL/6J mice (Jackson Laboratory) were crossed with *Fabp6* ^−/−^ mice (Praslickova et al., 2012) to produce an F1 generation of heterozygotes which were then inbred to yield *Fabp6^+/+^* and *Fabp6* ^−/−^ mice that were used in this study. Genotypes were confirmed by PCR using the primers described in Table S1. The age of the mice used in the study ranged from 12 to 28 weeks. Mice were housed (*n* ≤ 5 mice per cage) in a climate‐controlled facility with a reversed 12 h light:dark photoperiod. Mice were maintained on the Teklad 2020X diet (Teklad‐Envigo), which was used as the reference low‐fat diet (LFD). Age‐matched, *Fabp6^+/+^* and *Fabp6 Fabp6* ^−/−^ sibling mice (*n* = 8–12 per group; males and females in separate groups) were placed on the Western‐style diet (D12079B, Research Diets) for 10 weeks. For the LFD, the percentage of protein mass, total carbohydrate mass, and fat mass were 19.1%, 62% (47% available carbohydrates +15% fiber), and 6.5%, respectively. For the WSD, the percentage of protein mass, total carbohydrate mass, and fat mass were 19.8%, 55% (50% available carbohydrates +5% fiber), and 21%, respectively. Mice had free access to food and water. At the end of the controlled diet period, mice were fasted for 16 h prior to euthanasia by isoflurane/CO_2_ inhalation. Blood was collected by cardiac puncture into EDTA‐containing tubes and plasma was separated by centrifugation then stored at −70°C along with the tissues and gallbladder bile collected at necropsy. The use of animals in this study was approved by the animal care committee at McGill University in accordance with the Canadian Council of Animal Care guidelines.

### 2.2 Biochemical Assays

Total triacylglycerols and total cholesterol concentrations in the plasma were measured using commercial diagnostic assay kits (Wako Chemicals USA). Plasma lipid profile was determined by size exclusion chromatography using a Superose 6 column (GE Healthcare Life Sciences) attached to a Beckman System Gold HPLC system. For measurement of bile acid and fat excretion rates, mice were placed in individual cages and stools were collected daily then dried in a fume hood. Stools were pulverized into a fine powder in a Mini‐Beadbeater apparatus (BioSpec Products) using 2.3 mm stainless steel ball bearings as the grinding medium. Total bile acids were extracted from known mass of powdered stool samples using 50% *t*‐butanol (Van der Meer et al., 1985); and then quantitated using a commercial colorimetric enzymatic assay kit (Diazyme Bile Acids). Bile acids in gallbladder bile were identified and quantitated by HPLC as described previously (Torchia et al., 2001). Total lipids were extracted from a known mass of liver and powdered stool samples using previously described methods (Folch et al., 1957; Kraus et al., 2015).

### 2.3 Tracer experiments

In the first experiment, male and female mice (*n* = 3) of both genotypes, wild‐type and *Fabp6* ^−/−^, were orally gavaged with olive oil containing 0.05 g/ml of [1‐^13^C]palmitic acid (Sigma Chemical Co.) at 6 ml/kg body weight, housed individually with free access to LFD and water. Stools were collected after 24 h and then stored at −20°C until analysis. In the second experiment, WSD with [U‐^13^C]palmitic acid (CCLM409; Cambridge Isotope Laboratories) (10 μmol/g diet) and was fed over 3 days to individually housed wild‐type and *Fabp6* ^−/−^ mice (*n* = 3) of both sexes. Stools were collected every 24 h and stored at −20°C until analysis. Pentadecanoic acid (1 μmol) was added to each stool sample and fat was extracted as described above. Extracted fat was derivatized with pentafluorobenzyl bromide and N,N‐diisopropylethylamine overnight at 4°C. The derivatized fatty acids were extracted and reconstituted in hexane before gas chromatography mass spectrometry analysis (Agilent 5975C) using methane negative chemical ionization and select‐ion monitoring at *m/z* 256.4, *m/z* 257.4, and *m/z* 241.4 for the first experiment, and *m/z* 256.4, *m/z* 271.4, and *m/z* 241.4 for the second experiment.

### 2.4 Indirect calorimetry

Physical activity, energy expenditure, respiratory quotient, and food intake were measured using the Oxylet Metabolic Monitoring System (Panlab‐Harvard Apparatus). At the start of Week 9 of the controlled diet period, mice from each group were transferred to individual Physiocage apparatus for monitoring over 3 days, then returned to group housing after measurements. The total number of beam breaks in all axes (*x*, *y*, and *z* dimensions) represented the total physical activity. For both the energy expenditure and respiratory quotient, the areas under the curves were used to calculate statistics. The fat intake was estimated from food intake based on the fat content of each diet. Data were analyzed using GraphPad Prism version 8.21 (GraphPad Software Inc.).

### 2.5 Analysis of stool DNA

DNA was extracted from powdered stool samples following a previously published method by Tsai and Olson (1992). DNA concentration was determined fluorometrically using the DNA Qubit assay (Invitrogen). The number of total bacteria and bacteria belonging to Bacteroidetes, Firmicutes, and Proteobacteria phyla were estimated by quantitative PCR (Guo et al., 2008; Yang et al., 2015). The data are presented as relative bacterial load which is the ratio between each phylum and the total copy number (Navidshad et al., 2012). The murine single copy *Mos* gene (Gene ID: 17451) was used to measure the amount of murine DNA in the stool samples. In general, the extent of murine genomic DNA contamination in the stool samples was approximately 30 pg/µg of total stool DNA and was accounted for in the calculation of the gut microbial composition. The sequences of the primers used in this study are described in Table S1.

### 2.6 Statistics

Statistical analyses were performed using GraphPad Prism version 8.21 (GraphPad Software Inc.). Parametric and nonparametric tests were applied where appropriate. Data presented as mean ± SEM and means were compared using a two‐tailed unpaired Student's *t*‐test unless indicated otherwise. Differences were considered significant when *p* < 0.05.

## 3 RESULTS

### 3.1 Fat malabsorption is associated with bile malabsorption

**FIGURE 1:**
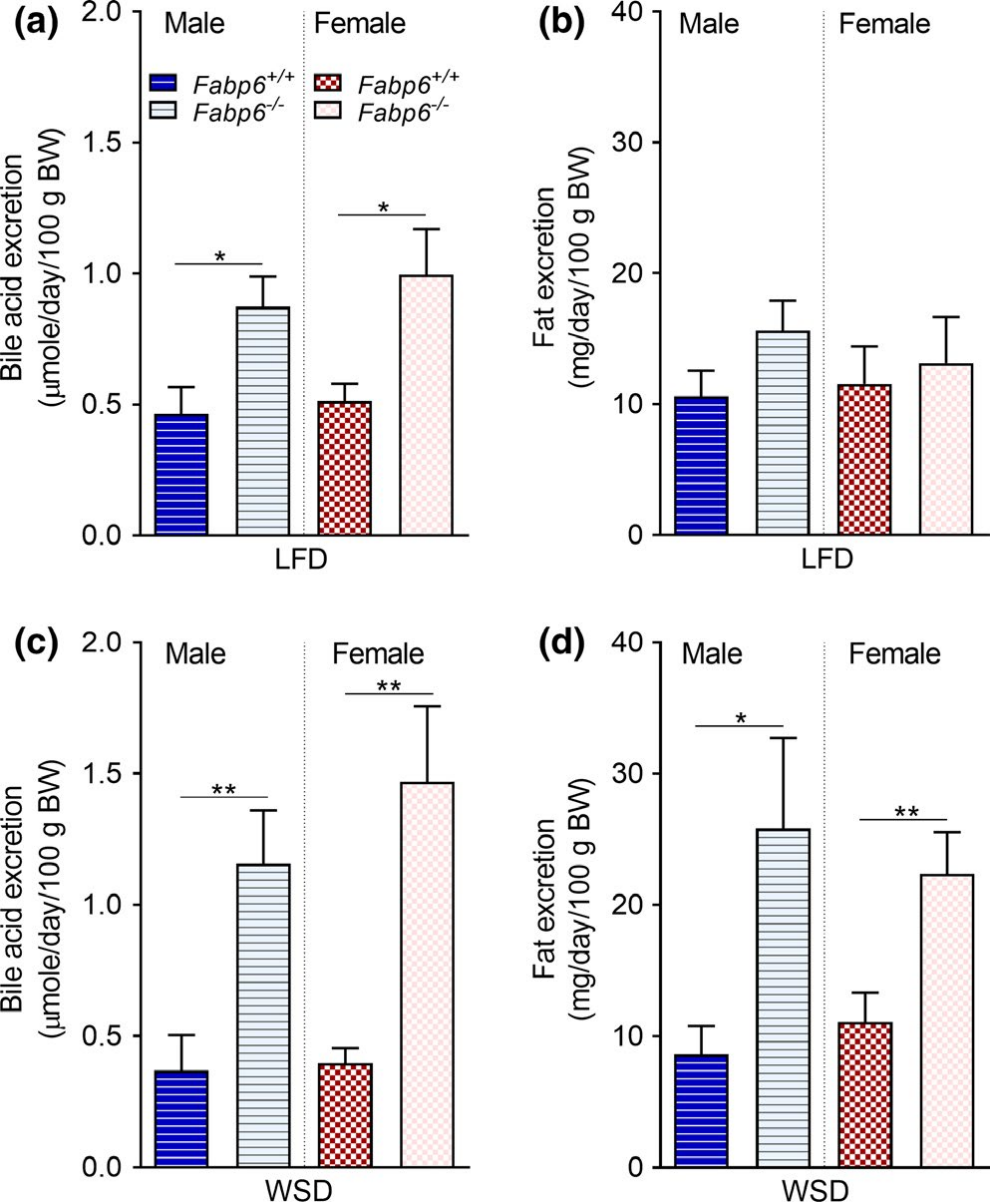
Fat malabsorption is associated with bile acid malabsorption. (a and c) Stool bile acid excretion (a and c), stool fat excretion (b and d) on the low‐fat diet (LFD) and Western‐style diet (WSD), respectively. The plot shows the combined data of two independent experiments. Dark and light bars indicate *Fabp6^+/+^* and *Fabp6^−/−^* mice, respectively. Mean ± SEM (*n* = 8–12 mice per group). Means were compared using Student's *t*‐test. *p < 0.05 and **p < 0.01

We previously showed that *Fabp6* ^−/−^ mice exhibit BAM on a diet with low‐fat content (Praslickova et al., 2012). To determine whether BAM causes fat malabsorption under high dietary fat diet intake, we fed wild‐type and *Fabp6* ^−/−^ mice the WSD for 10 weeks. Both male and female *Fabp6* ^−/−^ mice showed an increased rate of bile acid excretion compared to wild‐type mice on both the LFD (Figure 1a) and the WSD (Figure 1c). Concurrently, both male and female *Fabp6* ^−/−^ mice showed a higher total fat excretion rate compared to their wild‐type counterparts on the WSD (Figure 1d), but not on the LFD (Figure 1b). On both diets, fat intakes of male and female wild‐type and *Fabp6* ^−/−^ mice were comparable (Figure S1a,b). We also compared the total amount of fat excreted in stool and the total amount of dietary fat ingested. On the LFD, the total fat mass excreted, expressed as a percentage of total fat mass ingested, by both male and female *Fabp6* ^−/−^ mice was similar to that of wild‐type mice (Figure S1c). In contrast, on the WSD, it was higher for both male and female *Fabp6* ^−/−^ mice compared to that of their wild‐type counterparts (four‐ and twofold higher, respectively) (Figure S1d).

To confirm the correlation between BAM and fat malabsorption in *Fabp6* ^−/−^ mice we measured the amount of [1‐^13^C]palmitic acid excreted in stool during the 24 h following the delivery of the label by oral gavage, however, no change in excretion of the label by both male and female *Fabp6* ^−/−^ mice compared to wild‐type mice was observed (Figure S2a). Since differences in fat excretion between both genotypes were evident on WSD, we fed mice WSD mixed with [U‐^13^C]palmitic acid and measured the excretion of this label in stool over three days. Male *Fabp6* ^−/−^ mice excreted more label in the stool compared to male wild‐type mice (*p* < 0.05) (Figure S2b, left), however, no difference was observed between female *Fabp6* ^−/−^ and female wild‐type mice (*p* = 0.06; Figure S2b, right). Over time, the amount of excreted label in the stool of male and female *Fabp6* ^−/−^ mice increased whereas it decreased in both male and female wild‐type mice (Figure S2c). Based on these results, we concluded that the loss of Fabp6, which causes BAM (Praslickova et al., 2012) in both male and female mice, was associated with fat malabsorption when mice were fed a diet with a high dietary fat content.

### 3.2 The WSD induces greater adiposity in male *Fabp6* ^−/−^ mice than in male wild‐type mice

**FIGURE 2:**
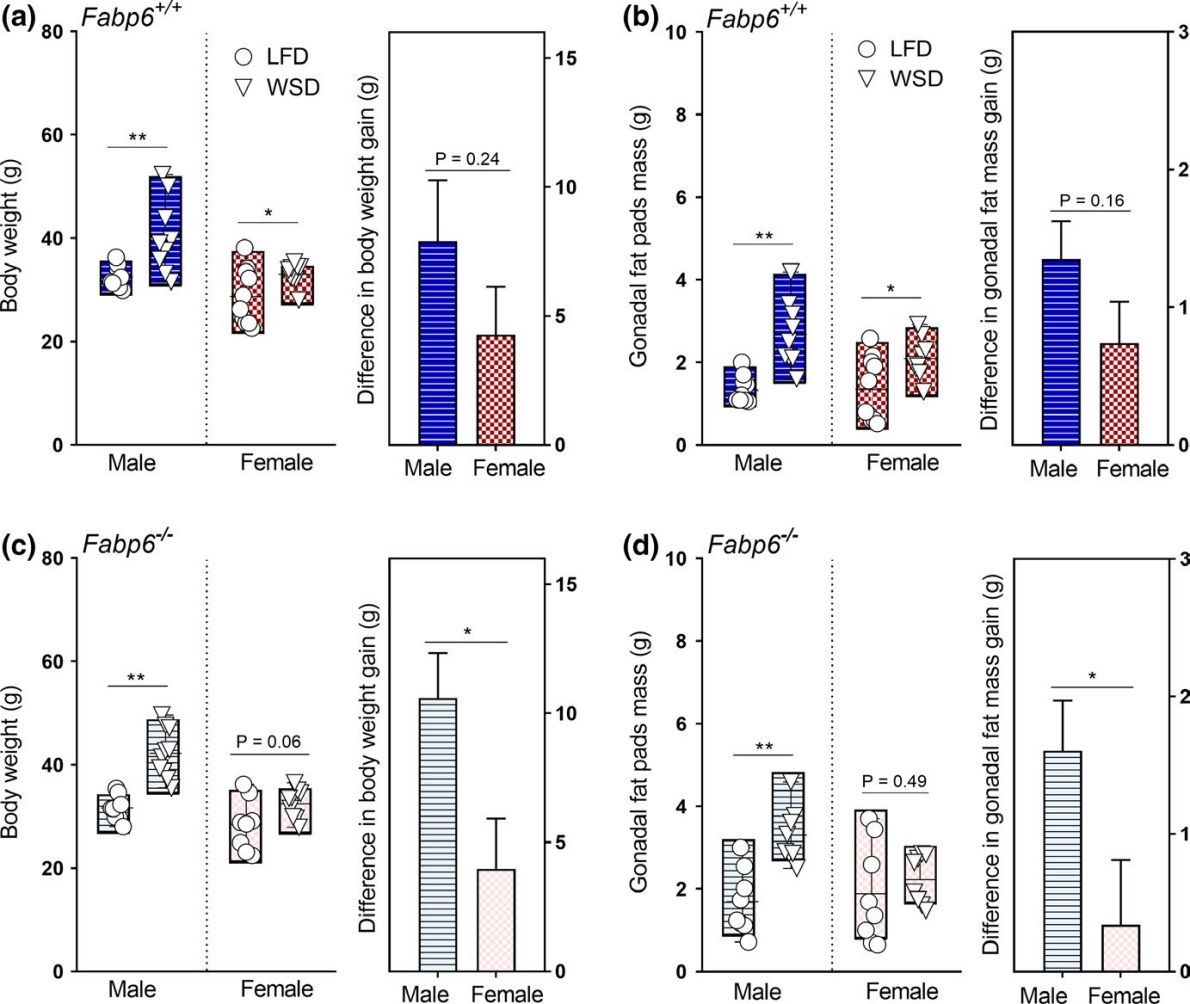
Greater adiposity in male *Fabp6^−/−^* mice on WSD. (a and c) Left, body weight; Right, difference in body weight gain of *Fabp6^+/+^* and *Fabp6^−/−^* mice on the low‐fat diet (LFD) and Western‐style diet (WSD), respectively. (b and d) Left, gonadal fat pads mass; Right, difference in gonadal fat mass gain of *Fabp6^+/+^* and *Fabp6^−/−^* mice on LFD and WSD, respectively. The plot shows the combined data of two independent experiments. Dark and light bars indicate *Fabp6^+/+^* and *Fabp6^−/−^* mice, respectively. Mean ± SEM (*n* = 8–12 mice per group). Means were compared using Student's *t*‐test. *p < 0.05, **p < 0.01

Interestingly, at necropsy, we noticed a higher degree of fat deposition in the viscera of male *Fabp6* ^−/−^ mice on the WSD compared to male wild‐type mice on the same diet while female *Fabp6* ^−/−^ mice did not show an obvious difference in the degree of fat deposition in the viscera compared to the female wild‐type mice. Comparison of gonadal fat pads revealed that male *Fabp6* ^−/−^ mice had greater increment in fat mass than female *Fabp6* ^−/−^ mice in response to the WSD (Figure 2d, right), a pattern that mirrored the increment in their body weights (Figure 2c, right). There was no difference between male and female wild‐type mice body weight or gonadal fat mass increments (Figure 2a,b, right). Thus, while male and female wild‐type mice had similar weight gain to their wild‐type counterparts in response to WSD, the deficiency of *Fabp6* caused greater adiposity in male mice than in female mice.

Since both male and female *Fabp6* ^−/−^ mice on WSD exhibited an increased rate of fat excretion compared to wild‐type mice, it seemed paradoxical that male *Fabp6* ^−/−^ mice gained more body weight and had greater adiposity than female *Fabp6* ^−/−^ mice. To gain insight into the metabolic response of male and female *Fabp6* ^−/−^ mice to the WSD, we first examined the plasma lipid concentrations of the mice. Total plasma cholesterol was higher in all mice on WSD compared to mice on the LFD (Table 1). Lipoprotein profile analysis showed higher low‐density lipoprotein cholesterol content in the plasma of male *Fabp6* ^−/−^ mice on WSD as compared to their female counterparts (Figure S3a,b, top). Male wild‐type and *Fabp6* ^−/−^ mice, but not female wild‐type and *Fabp6* ^−/−^ mice, showed higher total plasma triacylglycerol concentrations on the WSD compared to the LFD (Table 1). Male *Fabp6* ^−/−^ mice had higher very low‐density lipoprotein triacylglycerol on the WSD compared to the female mice (Figure S3a,b, bottom). Next, we measured total triacylglycerols and cholesterol concentrations in the liver. The WSD increased total liver cholesterol and triacylglycerols concentrations of male mice regardless of genotype (Table 1). Female wild‐type mice, but not female *Fabp6* ^−/−^ mice, had increased total liver cholesterol and triacylglycerols concentrations on the WSD (Table 1). Thus, the plasma and liver lipid profiles showed a sexually dimorphic response where male *Fabp6* ^−/−^ mice, but not female *Fabp6* ^−/−^ mice, showed elevations in plasma and liver cholesterol and triacylglycerols concentrations on the WSD. Taken together, the data demonstrated that the greater sensitivity of male *Fabp6* ^−/−^ mice to the WSD was associated with increased body weight gain and enhanced adiposity.

**TABLE 1:** Concentrations of total cholesterol and triacylglycerols in plasma and liver

	Male				Female			
	*Fabp6^+/+^*		*Fabp6* ^−/−^		*Fabp6^+/+^*		*Fabp6* ^−/−^	
	LFD	WSD	LFD	WSD	LFD	WSD	LFD	WSD
Plasma								
Total cholesterol (mmol/L)	1.95 ± 0.07	3.80 ± 0.27*	2.04 ± 0.12	3.69 ± 0.32*	2.09 ± 0.09	2.97 ± 0.16*	1.74 ± 0.14	2.77 ± 0.33*
Total triacylglycerol (mmol/L)	2.02 ± 0.04	3.22 ± 0.22*	1.87 ± 0.13	2.75 ± 0.14*	1.89 ± 0.14	2.19 ± 0.13	1.82 ± 0.10	2.12 ± 0.10
Liver								
Total cholesterol (mmol/L)	1.40 ± 0.04	1.64 ± 0.05*	1.39 ± 0.06	1.86 ± 0.13*	1.82 ± 0.07	2.11 ± 0.05*	2.15 ± 0.05	2.33 ± 0.04*
Total triacylglycerol (mmol/L)	4.73 ± 0.44	19.76 ± 5.1*	4.59 ± 0.24	23.23 ± 2.3*	5.55 ± 0.48	17.85 ± 2.9*	4.90 ± 0.67	15.57 ± 1.4*

Data presented as mean ± SEM (*n* = 4–8). Comparison between diets by unpaired Student's *t*‐test.

* *p* < 0.05.

### 3.3 Male *Fabp6* ^−/−^ mice are physically and metabolically active on the WSD

**FIGURE 3:**
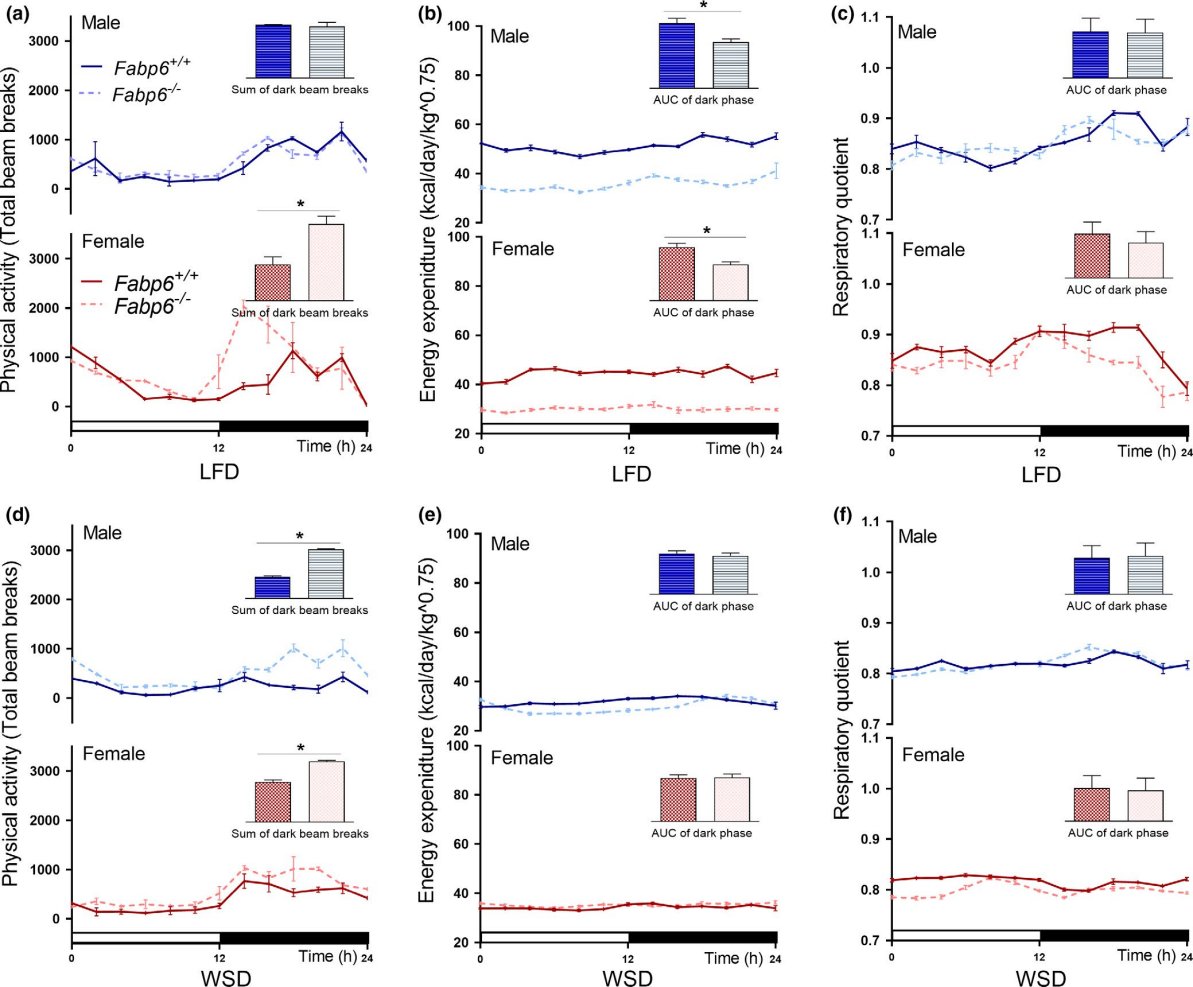
Enhanced physical activity and metabolic rate of male *Fabp6^−/−^* mice on WSD. (a and b) Rate of physical activity, (c and d) energy expenditure, (e and f) respiratory quotient on the reference low‐fat diet (LFD) and Western‐style diet (WSD), respectively. Data shown are representative of at least two independent experiments. The white bar represents the light period (12 h) and the black bar represents the dark period (12 h). *Fabp6^+/+^* mice are represented by the solid lines and dark bars, and *Fabp6* ^−/−^ mice are represented by broken lines and light bars. For each panel, top and bottom sections depict data from male and female mice, respectively. Insets represent the sum of beam breaks (*x*, *y*, and *z* axes) or area under the curve (AUC) during the dark phases as mean ± SEM (*n* = 6 mice per group) of three dark phases. Means were compared using Student's *t*‐test. *p < 0.05

To determine if the enhanced adiposity of male *Fabp6* ^−/−^ mice induced by the WSD was associated with altered metabolic rate, we measured the rate of physical activity and energy expenditure by indirect calorimetry. On the LFD, wild‐type mice displayed increased activity level during the dark phase compared to the light phase as expected (Figure 3a). On the WSD, male wild‐type mice showed a 60% reduction of activity level in the dark phase compared to wild‐type mice on the LFD (Figure 3a,b). In contrast, the physical activity level of male *Fabp6* ^−/−^ mice on the WSD was unchanged and remained comparable to that of wild‐type mice on the LFD (Figure 3a,b). Female wild‐type mice had similar levels of physical activity on both diets during the light and dark phases. In contrast, female *Fabp6* ^−/−^ mice had a lower activity level in the dark phase on the WSD compared to mice on the LFD (Figure 3a,b). Thus, the loss of *Fabp6* in mice also caused a sexually dimorphic effect on physical activity level, as male *Fabp6* ^−/−^ mice, but not female *Fabp6* ^−/−^ mice, remained physically active in response to the WSD.

Energy expenditure (EE) is a function of both physical activity and metabolic rate (Weir, 1949). Wild‐type mice had lower EE (40% lower in males; 20% lower in females) on the WSD compared to mice on the LFD regardless of sex in the dark phase (Figure 3c,d). Loss of Fabp6 also caused a reduction of EE on the LFD (Figure 3c). On WSD, male *Fabp6* ^−/−^ had lower EE (15%) compared to their wild‐type counterparts, whereas female *Fabp6* ^−/−^ mice had higher EE (15%) in the dark phase comapred to their wild‐type counterparts (Figure 3d). This reduction of EE in male *Fabp6* ^−/−^ mice on WSD was not comparable to that of male wild‐type mice on WSD and did not account for the enhanced adiposity displayed by the male *Fabp6* ^−/−^ mice.

Respiratory quotient (RQ) reflects the fuel substrate used for energy (Dewar & Newton, 1948). As expected, the RQ value for wild‐type mice consuming the WSD was lower than that for mice on the LFD (Figure 3e,f) regardless of sex. Interestingly, the RQ value for male *Fabp6* ^−/−^ mice on the WSD in the dark phase was greater than that observed for their wild‐type counterparts (Figure 3f). Female *Fabp6* ^−/−^ mice had a lower RQ value in the dark phase than female wild‐type mice on both diets (Figure 3e,f). Thus, the sex of *Fabp6* ^−/−^ mice modified the change in RQ values induced by the WSD, indicating that loss of Fabp6 affects substrate utilization in both male and female mice.

Together, these results demonstrate that the metabolic response of *Fabp6* ^−/−^ mice to the WSD was highly influenced by sex but the observed changes in physical activity and energy expenditure do not account for the enhanced adiposity in response to WSD feeding of male *Fabp6* ^−/−^ mice.

### 3.4 Alteration of gut microbiota in *Fabp6* ^−/−^ mice on WSD

**FIGURE 4:**
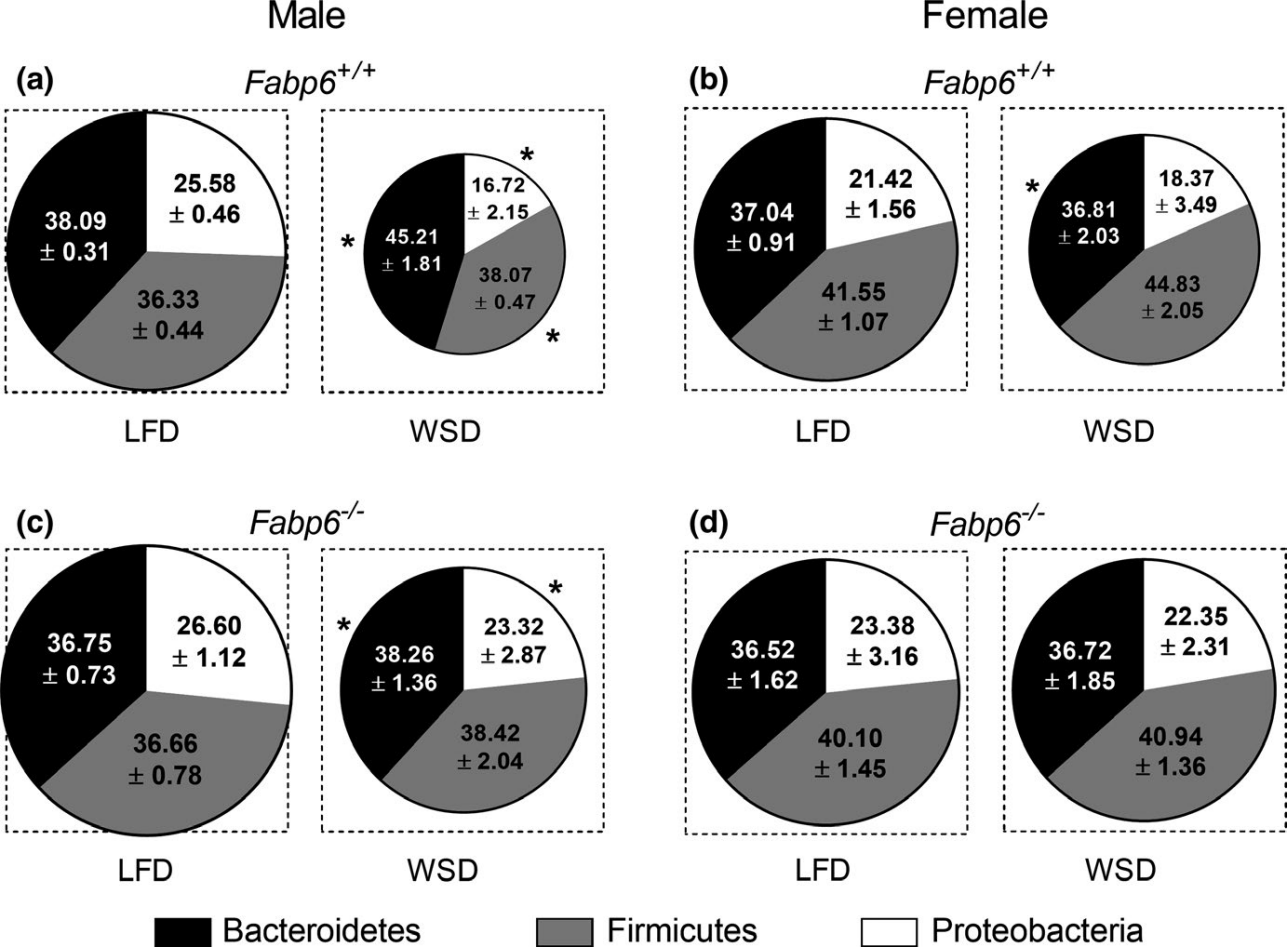
Remodeling of gut microbial composition. Gut microbial composition of male (a) and female (b) *Fabp6^+/+^* and *Fabp6^−/−^* mice (top and bottom, respectively). The area of the circles represents the percentage of total bacteria normalized to total number of bacteria determined for male *Fabp6^+/+^* mice, which was set to 100%. Data reported are representative of two independent experiments. Data represent the ratio between each phylum and the total copy number as mean ± SEM (*n* = 3–5). The dimensions of the boxes superimposed on the circles are equivalent. Bacteroidetes phylum shown in black, Firmicutes phylum in gray, and Proteobacteria phylum in white. The means of each phylum in the Western‐style diet (WSD) diet groups were compared to the corresponding phylum in the low fat diet (LFD) groups using Student's *t*‐test. *p < 0.05

Modulation of gut microbiota has been associated with changes in energy extraction efficiency from diet and consequently affect adiposity (Turnbaugh et al., 2006). Bile acids have bacteriostatic and bactericidal activity (Stacey & Webb, 1947; Watanabe et al., 2017) and thus have the potential to remodel gut microbiota. Since malabsorption of bile acids in the small intestine exposes the colonic bacteria to high concentrations of bile acids, we assayed for changes in the gut microbial community which is composed of the three main phyla, namely Bacteroidetes, Firmicutes, and Proteobacteria (Kostic et al., 2013). *Fabp6* ^−/−^ mice on the LFD showed similar total numbers of bacteria and phylum composition compared to wild‐type mice on the same diet (Males: Figure 4a,c; Females: Figure 4b,d). The feeding of the WSD diet decreased the total numbers of gut bacteria in both wild‐type male and female mice (by 27% and 12%, respectively; Figure 4a, top and Figure 4b, top), but expanded the Firmicutes and Bacteroidetes phyla at the expense of Proteobacteria phylum in male mice (Figure 4a,b) and reduced Bacteroidetes phylum in female mice (Figure 4b). In contrast to wild‐type mice, only male *Fabp6* ^−/−^ mice exhibited a decrease in the total number of gut bacteria in response to WSD, and this change was characterized by the expansion of the Bacteroidetes phylum at the expense of Proteobacteria phylum (Figure 4c, bottom).

We also evaluated the speciation of bile acids in the enterohepatic circulation by analyzing the composition of gallbladder bile. On the LFD, the loss of Fabp6 increased the concentration of secondary bile acids taurodeoxycholic acid (TDCA) in males (Figure S4a) and tauroursodeoxycholic (TUDCA) in females (Figure S4c), however, the ratio of taurocholic acid (TCA) to tauromuricholic acid (TMCA) was not affected in both sexes (Males: Figure S4a, right; Females: Figure S4c, right). This indicated that the speciation of bile acids in the enterohepatic circulation was altered by the loss of Fabp6. On the WSD, TDCA was higher in male *Fabp6* ^−/−^ mice (Figure S4b), whereas TCDCA, a primary bile acid, was higher in female *Fabp6* ^−/−^ mice (Figure S4d). The TCA:TMCA ratio was higher in the bile of male *Fabp6* ^−/−^ mice (Figure S4b, right) but not in female *Fabp6* ^−/−^ mice (Figure S4d, right). The observed change in bile acid speciation was in support of the observed modification in gut microbial composition. Moreover, the loss of Fabp6 in male mice, but not in female mice, resulted in the remodeling of the gut microbial community in response to the WSD to a pattern that is associated with enhanced adiposity.

## 4 DISCUSSION

Bile acids are essential for the digestion and absorption of dietary lipids (Ishibashi et al., 1996; Zwicker & Agellon, 2013). In humans, a mutation in the *SLC10A2* gene that encodes defective ASBT causes primary BAM and chronic steatorrhea (Heubi et al., 1982; Oelkers et al., 1997). Mice deficient in Asbt also show BAM, but unlike humans, fat malabsorption is not associated with BAM in this model (Dawson et al., 2003). The dissimilarity in phenotype could be attributable to differences in metabolism of bile acid between humans and mice (Agellon, 2008), or related to other factors that influence the overall efficiency of dietary fat absorption in these two species. We previously showed that deletion of the *Fabp6* gene in mice causes BAM (Praslickova et al., 2012). In this study, we studied *Fabp6* ^−/−^ mice to determine if BAM impacts fat dietary fat absorption. On the LFD, *Fabp6* ^−/−^ mice do not exhibit fat malabsorption; however, increasing the dietary fat content to a level that is normally found in the WSD provoked the significant reduction in efficiency of dietary fat absorption in both sexes of Fabp6‐deficient mice as compared to that of wild‐type mice on the same diet. Moreover, while efficiency of fat absorption was increased over time in wild‐type mice, it decreased over time in *Fabp6* ^−/−^ mice. This indicates the importance of Fabp6 in the regulation of fat absorption on high‐fat diets.

Unexpectedly, we discovered that male *Fabp6* ^−/−^ mice experienced enhanced adiposity in response to the WSD feeding compared to male wild‐type mice whereas the extent of adiposity induced by the WSD in female *Fabp6* ^−/−^ mice and female wild‐type mice was similar. The role of estrogen in regulating energy homeostasis and adiposity has been well established through inactivation of the *Cyp19* (encodes aromatase) and *Esr1* (encodes estrogen receptor α) genes in mice (Heine et al., 2000; Jones et al., 2000). However, an enhanced body weight gain phenomenon was also noticed in male *Slc51a* ^−/−^ mice (deficient in Ostα) compared to wild‐type mice fed the WSD (Hammond et al., 2015). It is more likely that the enhanced adiposity manifested by male *Fabp6* ^−/−^ mice is a consequence of disrupted bile acid metabolism in the gut caused by the loss of Fabp6, although estrogen may mask this effect since female *Fabp6* ^−/−^ mice did not show the same phenotype as male *Fabp6* ^−/−^ mice.

In this study, both the food intake and changes in metabolic rate induced by the WSD feeding did not account for the sex differential fat accretion shown by male *Fabp6* ^−/−^ mice. In addition, male *Fabp6* ^−/−^ mice fed the WSD, unlike female *Fabp6* ^−/−^ mice, maintained a high level of activity and a high rate of energy expenditure. Interestingly, in humans, the consumption of the WSD has been correlated with lower physical activity levels and sedentary behavior (Bibiloni et al., 2017; Ekelund et al., 2017). Our findings therefore suggest the possibility that bile acid metabolism may play a role in determining the level of physical activity.

Several studies have described the association between high Firmicutes to Bacteroidetes ratio and efficiency of energy extraction from the diet, which consequently leads to obesity (Backhed et al., 2004; Ley et al., 2005; Turnbaugh et al., 2006). Our findings suggest that exposure of the gut microbiota in *Fabp6* ^−/−^ mice to excess bile acids induced dysbiosis. Indeed, the appearance of secondary bile acids in gallbladder bile is consistent with the change in gut microbial composition. It has been determined that wild‐type male and female mice possess distinct gut microbiomes (Org et al., 2016). The expansion of the Firmicutes phylum we observed in male *Fabp6* ^−/−^ mice fed the WSD at the expense of other phyla is also distinct from that of female *Fabp6* ^−/−^ mice fed the same diet, and this is concordant with the pattern of change known to promote obesity (Musso et al., 2011; Ridaura et al., 2013). The selection of the WSD as the lipid‐rich diet in this study also exposed the sexually dimorphic response of *Fabp6* ^−/−^ mice to this diet, possibly due to its refined carbohydrate content. However, the specific nature of the taxonomic differences between male and female gut microbiomes that were induced by the exposure of the gut microbiota to the components of the WSD is not apparent from this study, but this should become evident by analysis of the changes in gut metagenome in future studies.

In summary, our study found that malabsorption of dietary fat in *Fabp6* ^−/−^ mice can occur coincidentally with BAM in the murine species but this is dependent on dietary fat content. Fortuitously, we uncovered a sexually dimorphic enhancement of adiposity in male *Fabp6* ^−/−^ mice as well as sexually distinct remodeling of the gut microbial composition in response to WSD feeding. This manuscript has supplementary files with supporting data.

## CONFLICT OF INTEREST

None declared.

## AUTHOR CONTRIBUTIONS

SMH and BLZ designed and performed the experiments, analyzed the data, and wrote the manuscript. LW oversaw the labeled palmitate experiments and edited the manuscript. LBA developed the overall study design, analyzed the data, wrote and edited the manuscript.

## Supporting information



Fig S1

Fig S2

Fig S3

Fig S4

Table S1

Table S2
