# Supplementary material for: Sexually dimorphic response of mice to the Western‐style diet caused by deficiency of fatty acid binding protein 6 (Fabp6)
Source: Physiol Rep. 2021 Feb 1;9(3):e14733. doi: 10.14814/phy2.14733 (PMC7851434; doi:10.14814/phy2.14733)
Supplement: Supplementary file 1 — Fig S1 [file PHY2-9-e14733-s001.pdf]

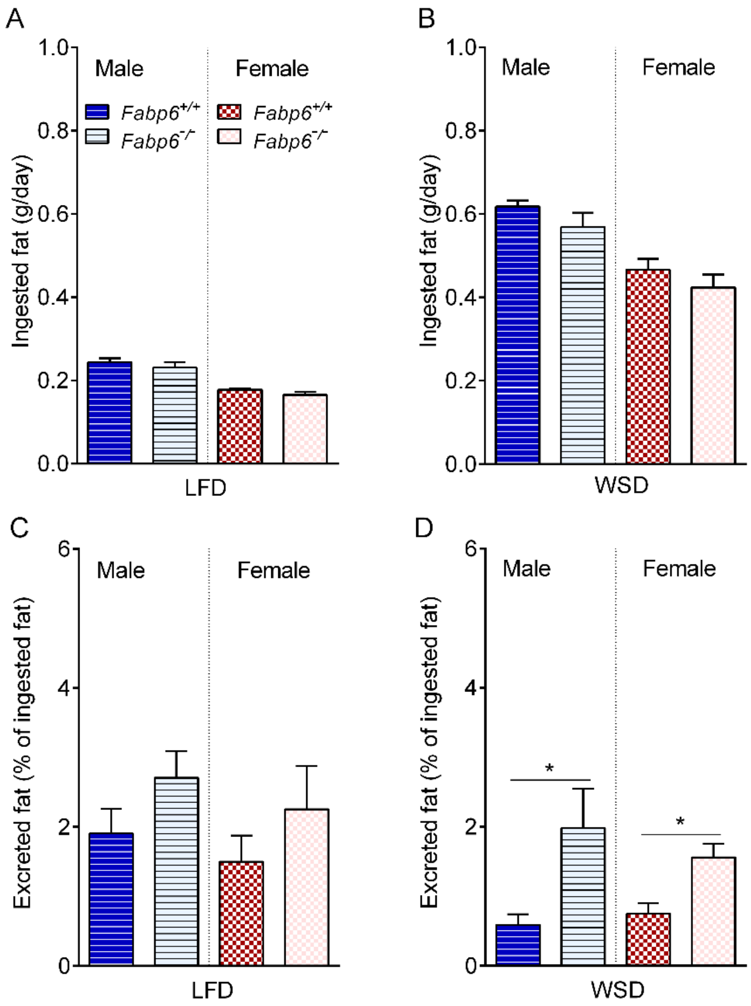

Fig. S1. Ingested fat and excreted fat. (A) and (B), mass of fat ingested per day (24 h) (C) and (D), mass of fat excreted into stool expressed as a percentage of mass of fat ingested. The mass of ingested fat (mean± SEM) was based on the mass of ingested food measured using the Oxylet Metabolic Monitoring System. Blue and red bars depict male and female mice, respectively. Dark and light bars indicate *Fabp6*$^{+/+}$ and *Fabp6*$^{-/-}$ mice (n=3 mice per group), respectively. Means were compared using Student's t-test and differences were considered statistically significant when *P<0.05. LFD, reference low fat diet. WSD, Western-style diet.
