# Supplementary material for: Sexually dimorphic response of mice to the Western‐style diet caused by deficiency of fatty acid binding protein 6 (Fabp6)
Source: Physiol Rep. 2021 Feb 1;9(3):e14733. doi: 10.14814/phy2.14733 (PMC7851434; doi:10.14814/phy2.14733)
Supplement: Supplementary file 2 — Fig S2 [file PHY2-9-e14733-s002.pdf]

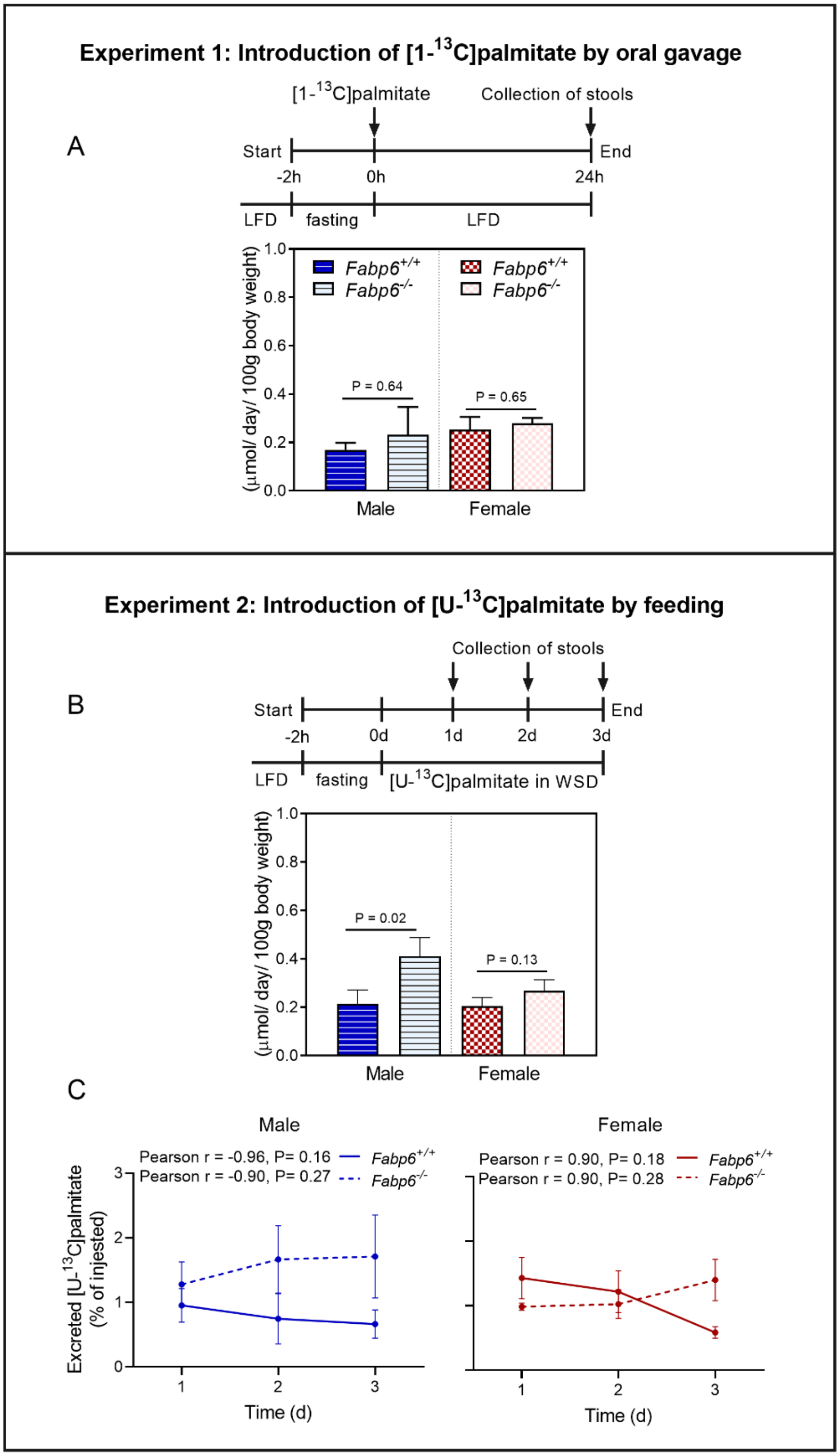

Fig. S2. Fate of orally administered palmitic acid. The experimental design and amount of (A) [1-^{13}C]palmitate and (B) [U-^{13}C]palmitate excreted into stool expressed as micromole per day per 100 g body weight (mean±SEM, n=3 mice per group). Blue and red bars depict male and female mice, respectively. Dark and light bars indicate *Fabp6*$^{+/+}$ and *Fabp6*$^{-/-}$ mice, respectively. (C) Mass of [U-^{13}C]palmitate excreted in stool over 3 days expressed as a percentage of mass of [U-^{13}C]palmitate ingested (mean±SEM, n=3 mice per group). *Fabp6*$^{+/+}$ mice are represented by the solid lines, and *Fabp6*$^{-/-}$ mice are represented by broken lines. Blue and red lines depict male and female mice, respectively LFD, reference low fat diet. WSD, Western-style diet.
