# Supplementary material for: Sexually dimorphic response of mice to the Western‐style diet caused by deficiency of fatty acid binding protein 6 (Fabp6)
Source: Physiol Rep. 2021 Feb 1;9(3):e14733. doi: 10.14814/phy2.14733 (PMC7851434; doi:10.14814/phy2.14733)
Supplement: Supplementary file 3 — Fig S3 [file PHY2-9-e14733-s003.pdf]

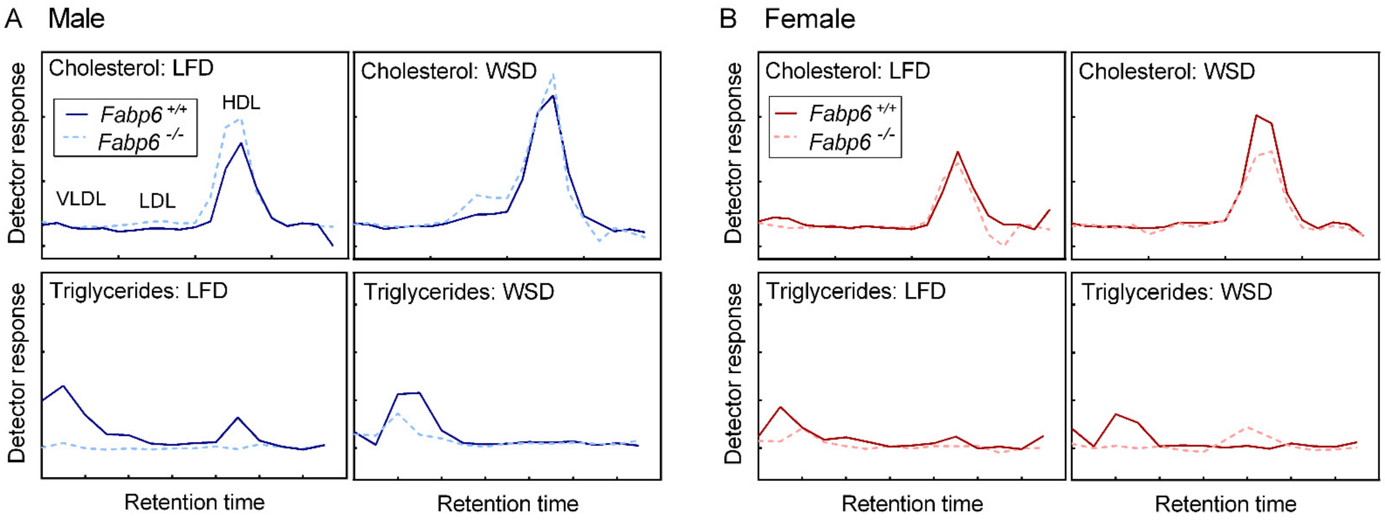

Fig. S3. Lipid profiles of plasma from (A) male and (B) female mice, respectively. The top and bottom panels depict cholesterol and triacylglycerol profiles, respectively. Samples from *Fabp6*$^{+/+}$ and *Fabp6*$^{-/-}$ mice are represented by a solid and broken lines, respectively. LFD, reference low fat diet. WSD, Western-style diet.
