# Supplementary material for: Sexually dimorphic response of mice to the Western‐style diet caused by deficiency of fatty acid binding protein 6 (Fabp6)
Source: Physiol Rep. 2021 Feb 1;9(3):e14733. doi: 10.14814/phy2.14733 (PMC7851434; doi:10.14814/phy2.14733)
Supplement: Supplementary file 4 — Fig S4 [file PHY2-9-e14733-s004.pdf]

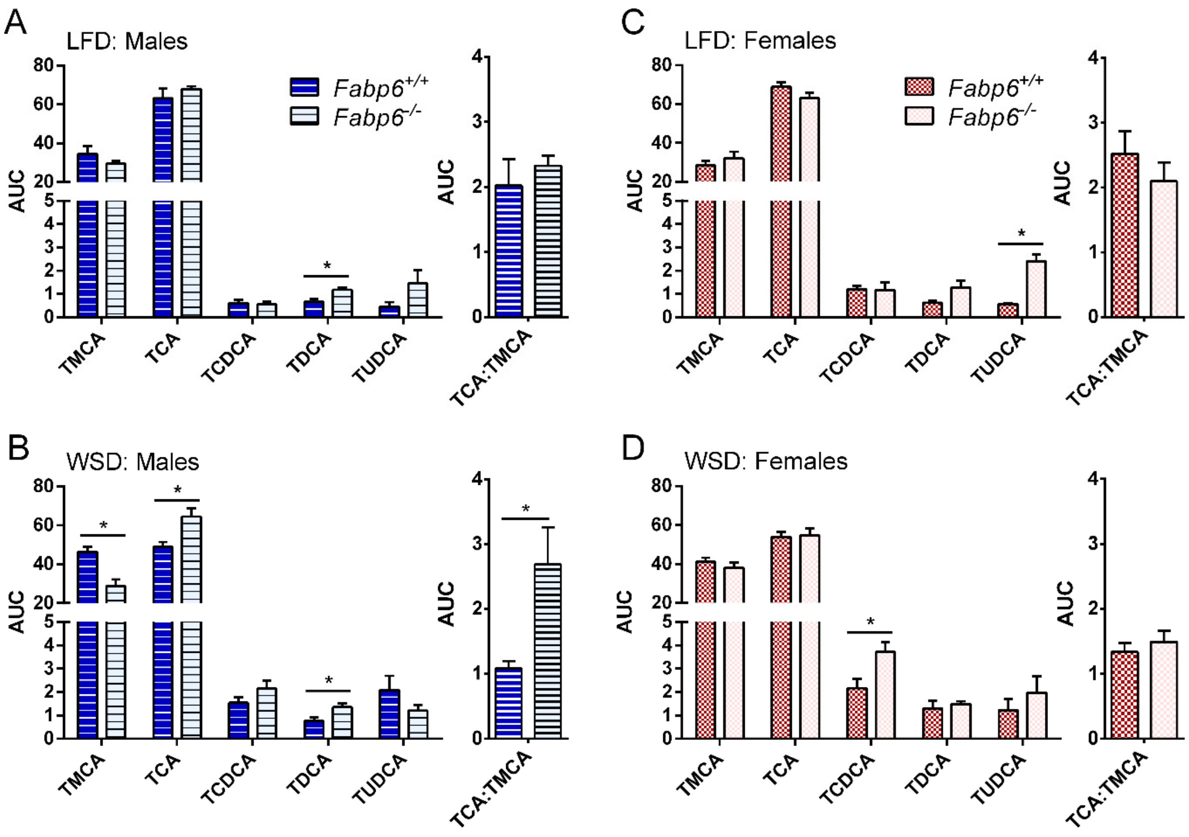

Fig. S4. Bile acid profiles of gallbladder bile from male (blue bars) and female (red bars) mice fed the reference low fat diet (LFD), (A) and (C), respectively, and the Western-style diet (WSD), (B) and (D), respectively. Mean±SEM (n=5-6 mice per group). Means were compared using Student's t-test and differences were considered statistically significant when *P<0.05. Dark bars, *Fabp6*$^{+/+}$ mice. Light bars, *Fabp6*$^{-/-}$ mice.
