# Supplementary material for: Sexually dimorphic response of mice to the Western‐style diet caused by deficiency of fatty acid binding protein 6 (Fabp6)
Source: Physiol Rep. 2021 Feb 1;9(3):e14733. doi: 10.14814/phy2.14733 (PMC7851434; doi:10.14814/phy2.14733)
Supplement: Supplementary file 5 — Table S1 [file PHY2-9-e14733-s005.pdf]

Table S1. Primer sequences.

Target	Direction	Primer sequence	Size (bp)
Fabp6 wildtype allele	Forward<br>Reverse	5'-ATGTGGAGCAGCAGGTTGTG<br>5'-TTACGCGCTCATAGGTCACA	449
Fabp6 disrupted allele	Forward<br>Reverse	5'-GACTGTGCCTTCTAGTTGCCA<br>5'-TTACGCGCTCATAGGTCACA	228
Bacteroidetes 16S rRNA[1]	Forward<br>Reverse	5'-GTTTAATTCGATGATACGCGAG<br>5'-TTAASCCGACACCTCACGG	122
Firmicutes 16S rRNA[2]	Forward<br>Reverse	5'-GGAGYATGTGGTTTAATTCGAAGCA<br>5'-AGCTGACGACAACCATGCAC	126
ε-proteobacteria 16S rRNA[1]	Forward<br>Reverse	5'-TAGGCTTGACATTGATAGAATC<br>5'-CTTACGAAGGCAGTCTCCTTA	109
Mouse *Mos* gene	Forward<br>Reverse	5'-ACATAAAGCATTGAGGTGCTAACAA<br>5'-TCAAAGTTCACCAAACTCCAGGT	102

[1]Yang YW, Chen MK, Yang BY et al. Use of 16S rRNA gene-targeted group-specific primers for real-time PCR analysis of predominant bacteria in mouse feces. Appl Environ Microbiol 2015;81;6749-6756.

[2]Guo X, Xia X, Tang R et al. Development of a real-time PCR method for Firmicutes and Bacteroidetes in faeces and its application to quantify intestinal population of obese and lean pigs. Lett Appl Microbiol 2008;47: 367-373.
