# Supplementary material for: Sexually dimorphic response of mice to the Western‐style diet caused by deficiency of fatty acid binding protein 6 (Fabp6)
Source: Physiol Rep. 2021 Feb 1;9(3):e14733. doi: 10.14814/phy2.14733 (PMC7851434; doi:10.14814/phy2.14733)
Supplement: Supplementary file 6 — Table S2 [file PHY2-9-e14733-s006.pdf]

Table S2. Fat, fatty acid and cholesterol content of the diets.

Component	Low fat diet[1] (g/Kg diet)	Western-style diet[2,3] (g/Kg diet)
Total saturated fatty acids	8	125
Total monounsatuarated fatty acids	11	60
Total polyunsatuarated fatty acids	29	13
Palmitic acid	6	53
Oleic acid	11	53
Linoleic acid	26	10
Linolenic acid	3	3
Stearic acid	1	24
Cholesterol	0	2

[1]Teklad global soy-protein free extruded rodent diet (2920X), Teklad-Envigo, Lachine, QC.

[2]Rodent western diet (D12079B), Research Diets, New Brunswick, NJ.

[3]Calculated using FoodData central (FDC ID 173412 and 748323): U.S. Department of Agriculture-Agricultural Research Service. (2019). FoodData central. Retrieved from fdc.nal.usda.gov.
